# Preclinical Efficacy of the Estrogen Receptor Degrader Fulvestrant in Combination with RAF/MEK Clamp Avutometinib and FAK Inhibitor in a Low-Grade Serous Ovarian Cancer Animal Model with Acquired Resistance to Chemotherapy and Aromatase Inhibitor

**DOI:** 10.3390/ijms26188924

**Published:** 2025-09-13

**Authors:** Cem Demirkiran, Stefania Bellone, Victoria M. Ettorre, Miranda Mansolf, Tobias Max Philipp Hartwich, Blair McNamara, Michelle Greenman, Yang Yang-Hartwich, Elena Ratner, Niccoló G. Santin, Namrata Sethi, Luca Palmieri, Silvia Coma, Jonathan A. Pachter, Sarah Ottum, Alessandro D. Santin

**Affiliations:** 1 Department of Obstetrics, Gynecology, and Reproductive Sciences, Yale University, New Haven, CT 06520, USA; cem.demirkiran@yale.edu (C.D.); stefania.bellone@yale.edu (S.B.); victoria.ettorre@yale.edu (V.M.E.); miranda.mansolf@yale.edu (M.M.); tobias.hartwich@yale.edu (T.M.P.H.); blair.mcnamara@yale.edu (B.M.); michelle.greenman@yale.edu (M.G.); yang.yang@yale.edu (Y.Y.-H.); elena.ratner@yale.edu (E.R.); niccol.santin@yale.edu (N.G.S.); namrata.sethi@yale.edu (N.S.); luca.palmieri@yale.edu (L.P.); sarah.ottum@yale.edu (S.O.); 2 Verastem Oncology, 117 Kendrick Street, Suite 500, Needham, MA 02494, USA; scoma@verastem.com (S.C.); jpachter@verastem.com (J.A.P.)

**Keywords:** low-grade serous ovarian cancer, fulvestrant, avutometinib, MEK inhibitor, FAK inhibitor

## Abstract

Low-grade-serous ovarian carcinomas (LGSOC) are rare tumors characterized by a high recurrence rate and limited treatment options. Most LGSOC are estrogen receptor (ER)-positive and demonstrate alterations in the RAS/MAPK pathway. Avutometinib is a dual RAF/MEK clamp, whereas defactinib and VS-4718 are focal adhesion kinase (FAK) inhibitors. Fulvestrant is an ER antagonist/degrader. We assessed the preclinical efficacy of fulvestrant, avutometinib + VS-4718 (FAKi), and the triple combination in a chemotherapy/aromatase inhibitor-resistant LGSOC patient-derived tumor xenograft (PDX) model. Tissue obtained from a LGSOC patient wild-type for KRAS/NRAS/BRAF mutations in progression after chemotherapy/anastrozole was transplanted into female CB17/lcrHsd-Prkdc/SCID mice (PDX-OVA(K)250). The animals were treated with either saline/control, fulvestrant, avutometinib/FAKi, or the triple combination of avutometinib/FAKi/fulvestrant. Avutometinib and FAKi were given five-days on and two-days off through oral gavage. Fulvestrant was administered subcutaneously weekly. Mechanistic studies were performed ex vivo using Western blot assays. Animals treated with the triple combination demonstrated stronger tumor growth inhibition compared to all the other experimental groups including control/saline (*p* < 0.001), single-agent fulvestrant (*p* = 0.04 from day eight and onwards), and avutometinib/FAKi (*p* = 0.02 from day 18). Median survival for mice treated with saline/control was 29 days while mice in all other experimental groups were alive at day 60 (*p* < 0.0001). Treatment was well tolerated across all experimental treatments. By Western blot, exposure of OVA(K)250 to the triple combination demonstrated a decrease in phosphorylated MEK (p-MEK) and p-ERK levels. The addition of fulvestrant to avutometinib/FAKi is well tolerated in vivo and enhances the antitumor activity of avutometinib/FAKi in a LGSOC-PDX model with acquired resistance to chemotherapy/aromatase inhibitors. These results support the clinical evaluation of avutometinib/defactinib in combination with fulvestrant or an aromatase inhibitor in patients with recurrent LGSOC.

## 1. Introduction

Low-grade serous ovarian cancer (LGSOC) is a rare, histopathologically, molecularly, and clinically distinct tumor from high-grade serous ovarian cancer (HGSOC) accounting for <10% of new epithelial ovarian tumors [1,2]. A review study published in 2023 reported that 34% of LGSOC were diagnosed at stage I-II, and the mean age of these patients was 59 years old [3]. Comprehensive surgical staging in early-stage disease and aggressive cytoreductive surgery in advanced stages or recurrent disease is the main treatment option for LGSOC [4,5]. While patients diagnosed with advanced LGSOC require adjuvant therapy subsequent to surgery, chemotherapy options have shown limited efficacy in LGSOC with an overall response rate (ORR) of 0–13% [6,7,8]. Development of novel, more effective treatment options for advanced/recurrent LGSOC patients are desperately needed.

A large number of LGSOC express estrogen receptor (ER) and harbor mutations in RAS/MAPK pathway genes including but not limited to KRAS, BRAF, and NRAS [9,10,11,12]. Specifically, a study published in 2022 reported that the rates of KRAS, BRAF, and NRAS gene mutations were 33%, 11%, and 8%, respectively [13]. Multiple reports have shown that LGSOC patients with RAS/MAPK pathway alterations have a better prognosis than LGSOC patients lacking RAS/MAPK pathway alterations [13,14]. The Gynecologic Oncology Group (GOG/NRG) in 2022 reported that the MEK1 and MEK2 allosteric inhibitor trametinib had higher efficacy than standard chemotherapy in patients with recurrent LGSOC [7]. Consistent with these data, Guo C et al. demonstrated the antitumor activity, safety, and toxicity profile of the oral RAF/MEK clamp avutometinib in LGSOC [15]. Based on these compelling clinical findings, it became evident that targeting the RAS/MAPK pathway downstream with RAF and MEK inhibitors holds substantial promise for improving the oncological outcomes in these patients.

Focal Adenosine Kinase (FAK) is a cytoplasmic tyrosine kinase with a vital role in cancer cell migration, proliferation, progression, and survival in multiple human tumors [16]. More importantly, FAK activation has been shown to represent a key adaptive resistance mechanism to the targeting of the RAS/MAPK pathway [17,18]. Accordingly, it may represent a promising therapeutic target in LGSOC patients, in particular when used in combination with RAS/MAPK pathway targeting. Importantly, preclinical studies showing the potent and synergistic antitumor activity of RAF/MEK clamp avutometinib and FAK inhibitor in xenograft mouse models with low-grade ovarian and carcinosarcoma have recently been reported by our research group [19,20].

Secondary to the high levels of estrogen receptor (ER) expression detected in the majority of LGSOC [12], anti-estrogen therapy, in particular, aromatase inhibitors (anastrozole, letrozole, and exemestane), are commonly recommended as alternative treatments to chemotherapy or following the use of cytotoxic regimens to a large number of LGSOC patients with recurrent disease [21]. In several studies, hormonal maintenance therapy following chemotherapy in patients with LGSOC has been reported to significantly improve median PFS compared to chemotherapy alone [22] and many studies evaluating the efficacy of a variety of hormonal therapies in women with LGSOC (MATAO trial NCT04111978, LEPRE trial NCT05601700, and NRG-GY-019 NCT04095364) are currently ongoing. Unfortunately, many LGSOC patients are either not responsive or eventually developed resistance to aromatase inhibitors treatment.

In this study, we took advantage of a PDX model developed from a patient with LGSOC who progressed after multiple lines of chemotherapy and the use of the aromatase inhibitor anastrozole to investigate the antitumor activity of the estrogen receptor degrader/antagonist fulvestrant in combination with the RAF/MEK clamp avutometinib and a FAKi (VS-4078/defactinib). Finally, we performed a Western blot assay to mechanistically evaluate the effect of this novel drug combination on the RAS/MAPK pathway.

## 2. Results

### 2.1. In Vivo Antitumor Activity of Avutometinib, VS-4718, and Fulvestrant Versus Control

**Figure 1:**
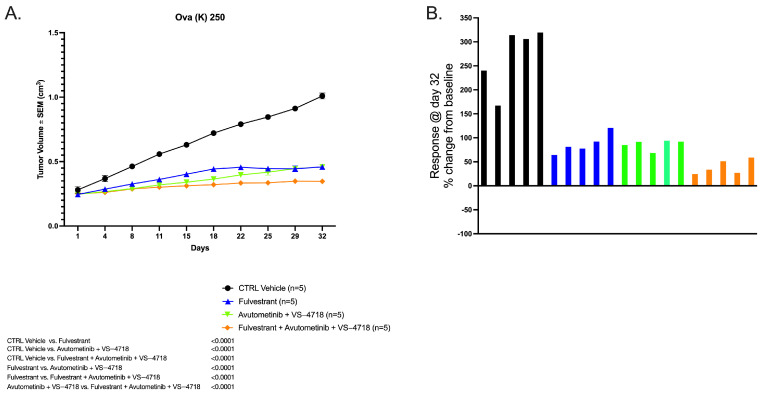
(**A**) Animals treated with the combination of avutometinib/FAKi (VS4718)/fulvestrant demonstrated significant tumor growth inhibition compared to controls from day 8 onwards, surpassing avutometinib/FAKi from day 18 and fulvestrant alone from day 8 (*p* < 0.0001, *p* = 0.02, *p*= 0.04, respectively) in OVA(K)250 PDX. Combination of all three drugs showed stable disease after day 22. (**B**) Percentage response change from baseline in individual animals treated with the different drugs.

The antitumor activities of an estrogen receptor antagonist/degrader (fulvestrant), a RAF/MEK clamp (avutometinib), and a FAK inhibitor (VS-4718) were determined by establishing xenografts from the primary LGSOC tumor sample OVA(K)250. This tumor xenograft exhibited consistent in vivo growth. Animals were randomly assigned into four different groups once tumor sizes reached 0.2 cm^3^. The combination of avutometinib, VS-4718, and fulvestrant was more effective in inhibiting tumor growth compared to control, fulvestrant single agent, and the combination of avutometinib plus VS-4718. Starting from day 8, we observed a significant difference in tumor growth inhibition between the treatment groups and vehicle control. On day 8, PDX OVA (K) 250 xenografts had mean tumor volumes of 0.28 cm^3^ (fulvestrant, avutometinib, and VS-4718 combination), vs. 0.29 cm^3^ (avutometinib plus VS-4718), vs. 0.32 cm^3^ (fulvestrant), and vs. 0.46 cm^3^ (vehicle control), respectively (*p* < 0.001 for the three-drug combination vs. control on day 8). Also, on day 8, the treatment group receiving the combination of three drugs had significantly smaller mean tumor volumes than the fulvestrant alone group (*p* = 0.04). On day 11, mice treated with the control vehicle had a mean tumor volume of 0.55 cm^3^ vs. 0.3 cm^3^ for those treated with fulvestrant/avutometinib/VS-4718 (*p* < 0.0001) (Figure 1A). Starting on day 18, the three-drug combination group had smaller mean tumor volumes than the dual combination of avutometinib plus VS-4718 (*p* = 0.02). After day 22, tumor growth had plateaued, and regression was observed with some animals in the treatment group receiving the triple combination of avutometinib, fulvestrant, and VS-4718. At the end of the study, we observed a clear difference in percentage response rate between the three-drug combination group and those receiving only fulvestrant or avutometinib/VS-4718 (Figure 1B).

**Figure 2:**
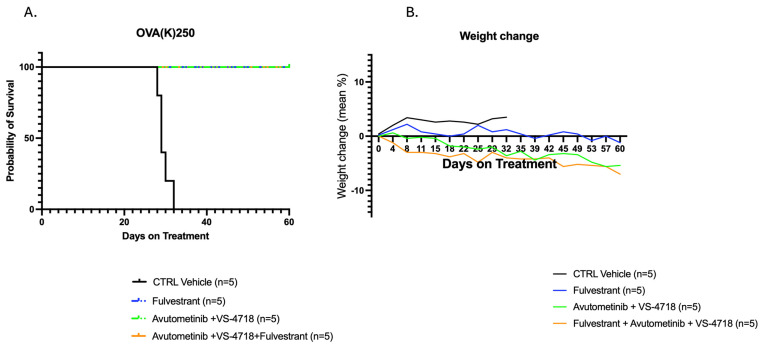
(**A**) Overall survival curves in fulvestrant, avutometinib + VS-4718, the combination of all three drugs, including avutometinib, fulvestrant and, VS-4718, and the control groups. All three groups exhibited a significant survival advantage over control. Median survival for the control group is 29 days, whereas the rest of the groups survived until the end of the treatment (*p* < 0.0001). (**B**) Mice tolerated all treatments well without significantly impacting body weight compared to the control group.

While the median survival for the vehicle control group was only 29 days, mice treated with fulvestrant alone, avutometinib + VS-4718, or the triple-drug regimen (avutometinib, VS-4718, and fulvestrant) survived until day 60. Overall survival (OS) difference curves between study groups receiving the combination of a three-drug, fulvestrant alone, avutometinib + VS-4718, and control were statistically significant (*p* < 0.0001) (Figure 2A). Assessing body weight loss is a crucial and reliable indicator of dose-limiting toxicity in in vivo therapy evaluations. Accordingly, the body weight of the four groups of mice was measured twice a week. None of the groups showed a mean body weight loss of 10% or more (Figure 2B).

### 2.2. Avutometinib, VS-4718, and Fulvestrant Treatment Inhibits ERK and FAK Activation in an Ex Vivo Model of LGSOC

**Figure 3:**
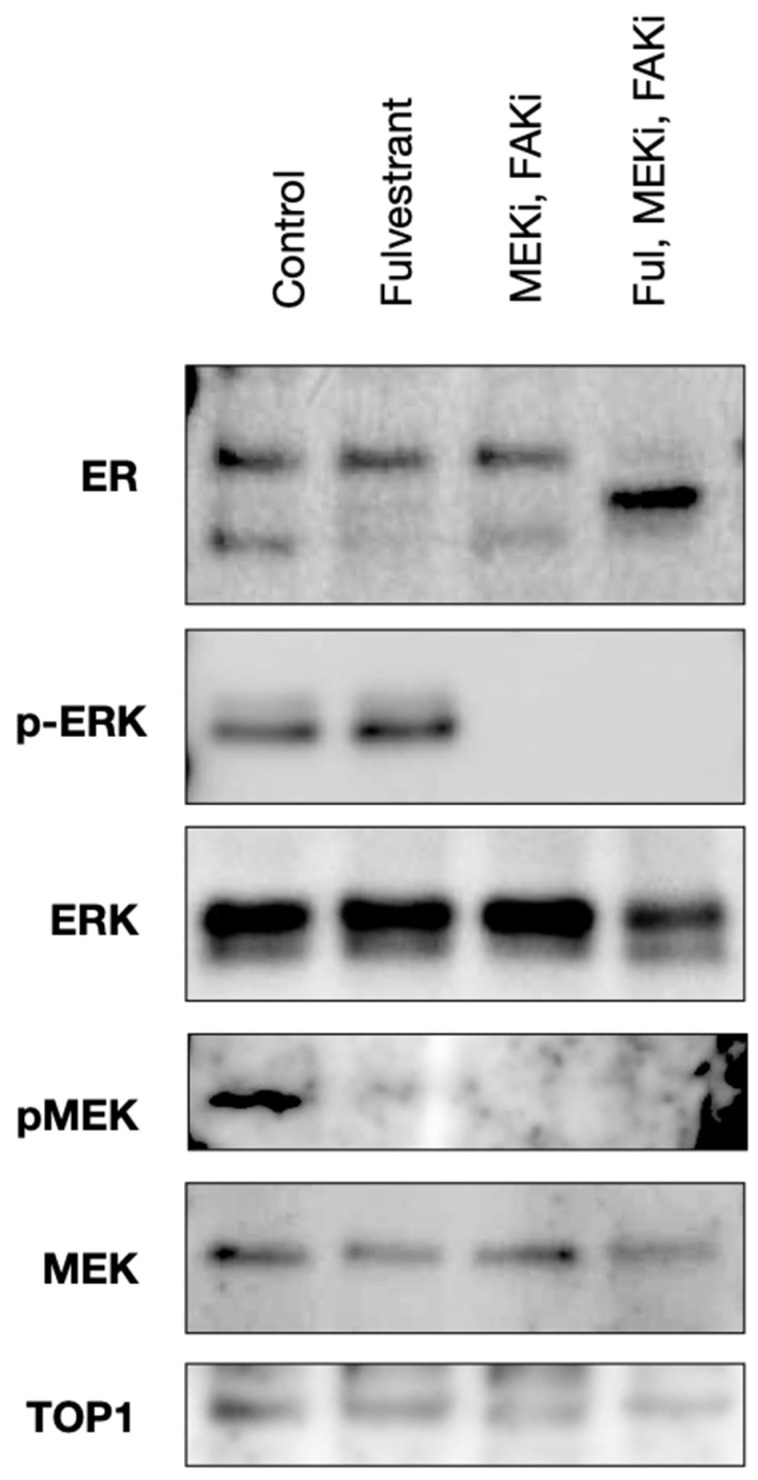
Western blot analysis of the LGSOC model OVA(K)250 treated ex vivo with fulvestrant, the dual combination of avutometinib + defactinib, and the triple-drug combination of fulvestrant/avutometinib/defactinib. The triple-drug combination reduced both p-MEK and p-ERK. Total MEK and ERK levels were mildly affected. Fulvestrant was also able to decrease p-MEK levels. The triple-drug combination led to a decrease in ER protein levels. Band Weights: ER 66 kDa, p-ERK 42 kDa, ERK 44 kDa, pMEK 45 kDa, MEK 45 kDa, and TOP1 100 kDa. Full blots and quantification available in Supplemental Materials.

We assessed the impact of avutometinib, defactinib, and fulvestrant on p-ERK, p-MEK, and estrogen receptor levels using Western blot analysis. The three-drug combination and the dual avutometinib + defactinib combination reduced both p-MEK and p-ERK without impacting total MEK and ERK levels after two hours of treatment in the ex vivo model compared to the untreated control (Figure 3). Fulvestrant was also able to decrease p-MEK levels. These results unequivocally confirm the activity of avutometinib and defactinib as potent inhibitors of the RAS/MAPK pathway and suggest that the ER degrader fulvestrant is also able to inhibit the RAS/MAPK pathway.

## 3. Discussion

LGSOC are rare tumors diagnosed at a younger age when compared to HGSOC patients [23]. While these patients tend to experience prolonged survival, LGSOC have lower sensitivity to standard chemotherapy protocols [24]. Accordingly, the development of novel, more effective treatment modalities for advanced/recurrent LGSOC patients remains an unmet medical need.

Targeting the RAS/MAPK pathway with MEKi is one of the newer treatment options for women with recurrent LGSOC. Consistent with this view, Shotton et al. assessed the effectiveness of the MEKi trametinib in the treatment of patients with recurrent LGSOC in a multicenter study [25]. They discovered that the rates of partial response, stable disease, and disease progression were 21%, 32%, and 36%, respectively. The GOG 281 phase 2/3 trial, which compared trametinib with standard chemotherapy of physician’s choice in the management of recurrent LGSOC, showed great potential to significantly impact patient care [7]. The authors reported that the median PFS was 13 months for trametinib versus 7.2 months for chemotherapy (*p* < 0.001), and the overall response rates for trametinib and chemotherapy groups were 28% and 5%, respectively. Median OS were 37.6 and 29.2 months for trametinib and chemotherapy groups, respectively (*p* = 0.056). Importantly, in this study, KRAS, BRAF, and NRAS mutation status did not affect PFS results, although KRAS, BRAF, and NRAS status were retrospectively assessed and only in approximately 50% of the patients in the study. In another phase 3 trial (i.e., MILO/ENGOT-ov11 trial), 341 patients with recurrent LGSOC were randomized 2:1 to the MEKi binimetinib or physician’s choice of chemotherapy [26]. The rate of total RAS/MAPK pathway gene mutations (KRAS, NRAS, BRAF V600E, RAF1, and NF1) in 135 patients treated with binimetinib was found to be 57.4%, and response rates in a post hoc analysis were 41% vs. 13% in RAS/MAPK genes mutation-positive and -negative groups, respectively. Also, PFS was significantly longer in the mutation-positive group compared to the mutation-negative group [HR, 0.5; 95% confidence interval (CI) 0.31–0.79]. However, these MEKi have not been widely adopted for treatment of recurrent LGSOC due to their toxicities, reflected by high discontinuation rates for adverse events with trametinib (36%) and binimetinib (31%).

In a recent study, we performed a preclinical evaluation of the RAF/MEK clamp avutometinib, alone and in combination with the FAK inhibitor VS-4718, in the fully characterized OVA(K)250 PDX model [19]. This study demonstrated that the combination of avutometinib plus VS-4718 has a strong tumor growth inhibition efficacy compared to controls starting at day 9 in OVA(K)250 PDX (*p* < 0.002). At the end of the study, mice treated with avutometinib alone and avutometinib plus VS-4718 remained alive, compared to median survival of 20 days in control mice and 35 days in VS-4718-treated mice (*p* < 0.0001). By Western blot assays, exposure of OVA(K)250 to avutometinib, the FAK inhibitor defactinib, and their combination demonstrated decreased phosphorylated FAK (p-FAK) as well as reduced p-ERK. These preclinical data are consistent with the results of a randomized, multicenter, open-label phase II study (i.e., ENGOT-ov60/GOG-3052/ RAMP 201) (NCT04625270) in recurrent LGSOC patients treated with avutometinib in combination with defactinib [27]. Study authors reported an overall response rate (ORR) of 31% in the overall population with an ORR of 44% in patients with KRAS mutations and an ORR of 17% in patients without KRAS mutations. Tumor shrinkage was observed in the vast majority (82%) of the LGSOC patients regardless of KRAS status. Median progression-free survival was 22 months in patients with KRAS-mutant LGSOC. Most of these patients had failed chemotherapy, hormonal treatment (mainly aromatase inhibitors), and/or prior MEKi treatment.

A large proportion of LGSOCs express estrogen receptors [12]. Accordingly, multiple studies have previously investigated the therapeutic effect of anti-estrogenic therapy in patients with LGSOC, with some demonstrating that anti-estrogen drugs may improve oncological outcomes [22]. Fader et al. investigated the therapeutic effect of adjuvant anti-estrogenic therapy alone (most patients received anastrozole or letrozole) following cytoreductive surgery in women with stage II-IV LGSOC. The authors reported that the two- and three-year PFS and OS were 82.8 and 96.3%, and 79.0 and 92.6%, respectively [28]. MATAO, an ongoing phase III clinical trial with letrozole into ER/PR positive with ovarian cancer patients [29] and NCT05113368, a phase II clinical trial with fulvestrant in combination with regorafenib, will soon provide additional insights about the clinical activity of these drugs in LGSOC patients.

In the present study, we took advantage of a LGSOC PDX model (i.e., OVA(K)250) recently established in our laboratory from a patient in progression after multiple lines of chemotherapy and aromatase inhibitors to evaluate the antitumor activity of the estrogen receptor antagonist/degrader (fulvestrant) used alone or in combination with the novel RAF/MEK clamp avutometinib and the FAKi VS-4718 used as surrogate for the FAK inhibitor defactinib. Starting on day eight of our in vivo experiment, a significant difference in tumor growth inhibition between the drug-treated groups vs. vehicle control was observed. On day 11, mice in the control vehicle group had a mean tumor volume of 0.55 cm^3^ vs. 0.3 cm^3^ for those treated with the three-drug combination (fulvestrant, avutometinib, and VS-4718) (*p* < 0.0001). Importantly, the triple combination of fulvestrant with avutometinib/VS-4718 provided better tumor control when compared to both single-agent fulvestrant or the dual combination of avutometinib/VS-4718. All drug-treated animals demonstrated prolonged survival when compared to the vehicle-group-treated animals which survived only 29 days (*p* < 0.0001). The dual and triple combinations were well tolerated in mice.

In previous studies, Western blot results demonstrated that the levels of p-ERK were reduced in LGSOC when exposed to avutometinib and defactinib, indicating that these drugs specifically target and significantly impact the RAS/MAPK pathway [19]. Since most advanced/recurrent LGSOC patients are exposed and eventually progress on chemotherapy and aromatase inhibitors and MEKi have been demonstrated to induce a feedback response that results in ERα overexpression, phosphorylation, and transcriptional activation of ER-regulated genes [23], we thought the use of an ER antagonist/degrader like fulvestrant may potentiate the clinical activity of the combination of avutometinib + FAKi. Accordingly, we investigated the potential mechanism behind the triple combination in vivo activity using Western blot experiment in OVA(K)250), a chemotherapy and aromatase inhibitor-resistant LGSOC model. We found the dual and triple combination to be able to not only decrease tumor growth in vivo but also reduce the active (i.e., phosphorylated) forms of MEK and ERK (i.e., p-MEK and p-ERK) in LGSOC cells by Western blot assays. These results are in agreement with previous data combining MEKi and fulvestrant in other human tumor models demonstrating that MEKi can be used to sensitize ERα-positive tumors to hormonal therapy, and accordingly, propose that this strategy may have broader clinical utility in other ERα-positive carcinoma [30]. Previous studies have shown that estrogen receptor degradation occurs via protease activity and can result in a range of degradation products on Western blot [31]. Similarly, a new band with slightly lower molecular weight was noted in our Western blot experiments after exposure of the LGSOC to the triple-drug combination. This band may represent the above-mentioned accumulation of ER degradation products. If so, this may represent a possible synergy between fulvestrant and MEK and FAK inhibitors, leading to increased ER degradation in ovarian tumors, which we may study in the future.

## 4. Materials and Methods

### 4.1. Specimen Collection and Formation of PDX Models

The study protocol was approved by the Yale Human Investigation Committee and was conducted in accordance with the Declaration of Helsinki. Prior to initiating any research activities, informed consent from patients and/or their legally authorized representatives was obtained. LGSOC tissue samples were obtained from clinically indicated biopsies performed at the time of tumor recurrence. Briefly, OVA (K) 250 was xenografted with Matrigel (Corning Life Sciences, Corning, NY, USA) into female CB17/lcrHsd-Prkdc/SCID mice into the subcutaneous tissue of the lower abdomen. Tumor size was assessed 2 to 3 times per week using Vernier calipers. Once mice showed consistent PDX growth, they were randomly assigned to treatment groups as outlined below.

### 4.2. Avutometinib, Defactinib, VS-4718, and Fulvestrant

Verastem, Inc. (Needham, MA, USA) supplied the following compounds—avutometinib, defactinib, and VS-4718—under a material transfer agreement. Fulvestrant was purchased from Northstar healthcare, Memphis, TN. VS-4718 is a focal adhesion kinase inhibitor (FAKi) frequently used in animal experiments as an experimental replacement for defactinib, the FAKi used in patients in the clinic.

### 4.3. In Vivo Treatment

Briefly, PDX mice were randomized into treatment groups when the tumor reached a volume of 0.2 cm^3^. There were three treatment groups, with 5 mice in each group: control group, combination group of avutometinib (RAF/MEK clamp) with VS-4718 (FAKi), Fulvestrant (estrogen receptor antagonist) group, and the combination of all three drugs avutometinib/VS-4718/fulvestrant. Fulvestrant was dosed at 5 mg per mouse subcutaneously once a week. Avutometinib (0.3 mg/kg once daily) and VS-4718 (50 mg/kg twice daily) were administered by oral gavage for five days with a subsequent two-day break. Mice were observed for overall survival (OS), and the size of the tumor was measured twice weekly. As a surrogate for toxicity assessment, the animal weight was collected twice weekly; if the animal’s weight dropped below 10% of their starting weight, treatment was held. If mice appeared to be poor in health, tumor became necrotic, or when tumor volume reached above 1.0–1.25 cm^3^ using the formula length × (width)^2^/2, mice were sacrificed. At the end of the study, the surviving mice were euthanized following the rules and regulations set forth by the Yale IACUC.

### 4.4. Ex Vivo Tumor Tissue Culture and Treatment

After tumor tissues were harvested from untreated OVA(K)250 PDX-bearing mice to ensure a uniform baseline without prior in vivo drug exposure, they were cut into 3 mm × 3 mm × 3 mm pieces. The decision was made to develop an ex vivo model to isolate tumors from mice and treat tumor pieces because these cell lines are difficult to grow in vitro and can only be propagated in vivo using PDX models. For each treatment group, we treated 5 individual tumor pieces in Petri dishes. Tumor pieces were incubated for 30 min in RPMI 1640 medium (Life Technologies, Grand Island, NY, USA) containing streptomycin/penicillin (Life Technologies) and 10% fetal bovine serum (Sigma-Aldrich, St. Louis, MO, USA) at 37 °C and 5% CO_2_. Tumors were then treated with DMSO solvent (0.1%) as untreated control, the combination of avutometinib and defactinib, fulvestrant, and finally with the triple combination of avutometinib, defactinib, and fulvestrant for 2 h. After treatment, tumor pieces were collected, homogenized using a BeadBug Homogenizer (Benchmark Scientific, Sayreville, NJ, USA) and used for preparing protein lysates.

### 4.5. Western Blot

Protein lysates of pooled tumor pieces were prepared from the same treatment group and homogenized using lysis buffer (1% Triton X-100, 0.05% SDS, 100 mM Na_2_HPO_4_, and 150 mM NaCl). Afterwards, lysates were electrophoresed on a 4–20% pre-cast SDS–polyacrylamide gel (Bio-Rad, Hercules, CA, USA) and transferred onto Amersham Hybond 0.45 PVDF membranes (GE Healthcare, Chicago, IL, USA). Membranes were then blocked for 60 min with 5% non-fat milk in PBS-0.05% Tween 20. After incubation with primary antibodies at 4 °C overnight, the membranes were then incubated with secondary antibodies for 1 h at room temperature. Antibodies include anti-MEK (#9122, Cell Signaling Technology, Danvers, MA, USA), anti-p-MEK (#9121, Cell Signaling Technology), anti-ERK (#9102, Cell Signaling Technology), anti-p-ERK (#4376, Cell Signaling Technology), topoisomerase 1 (TOP1) (BD Biosciences # 556597, Franklin Lakes, NJ, USA), and estrogen receptor alpha (D8H8) (#8644, Cell Signaling Technology). Blots were designed by using Clarity or Clarity Max Western ECL Blotting Substrates (Bio-Rad). TOP1 was used as a housekeeping control gene in these experiments. LICORbio Image Studio (version 6.1 was used to perform densitometry readings.

### 4.6. Statistical Analysis

Statistical analysis was performed using the GraphPad Prism version 10 (GraphPad Software, Inc. San Diego, CA, USA). Significance in tumor volumes at specific timepoints for in vivo experiments was determined using a two-way ANOVA. When the tumor in the individual mice exceeded 1.0 cm^3^ in measured volume or was ulcerated, treatment was halted, and the animal was euthanized. Overall survival (OS) data, defined as the time from enrollment to death, were analyzed and plotted using Kaplan–Meier survival curves. The curves were then compared using the log-rank test. All observed differences in the comparisons have been proven to be statistically significant at *p*-values of less than 0.05.

## 5. Conclusions

In this preclinical study, we demonstrated for the first time that the addition of fulvestrant to the active avutometinib/defactinib combination may significantly increase tumor control when compared to fulvestrant alone or compared to the combination of avutometinib plus defactinib in a KRAS wild-type LGSOC PDX model resistant to chemotherapy and aromatase inhibitor. Mechanistic studies demonstrated dephosphorylation in both ERK as well as MEK protein after exposure to the combination. These preclinical results are consistent with the encouraging data recently reported in the GOG-3052; NCT04625270; RAMP 201; and ENGOT-ov60 trial evaluating the clinical activity of the avutometinib/defactinib combination in recurrent LGSOC patients. Importantly, these results suggest that the addition of an ER degrader such as fulvestrant, or potentially an aromatase inhibitor, may further increase the activity of the combination of avutometinib plus defactinib in LGSOC patients with recurrent disease resistant to chemotherapy and aromatase inhibitor.

## Data Availability

Upon request, data is available.

## Supplementary Materials

The following supporting information can be downloaded at: https://www.mdpi.com/article/10.3390/ijms26188924/s1.

## Abbreviations

The following abbreviations are used in this manuscript:

LGSOC	Low-Grade Serous Ovarian Cancer
HGSOC	High-Grade Serous Ovarian Cancer
ORR	Overall response rate
FAK	Focal Adhesion Kinase
FAKi	Focal Adhesion Kinase Inhibitor
PDX	Patient-Derived Xenograft
OS	Overall Survival
IC	Confidence Interval
