# Supplementary material for: Preclinical Efficacy of the Estrogen Receptor Degrader Fulvestrant in Combination with RAF/MEK Clamp Avutometinib and FAK Inhibitor in a Low-Grade Serous Ovarian Cancer Animal Model with Acquired Resistance to Chemotherapy and Aromatase Inhibitor
Source: Int J Mol Sci. 2025 Sep 13;26(18):8924. doi: 10.3390/ijms26188924 (PMC12469703; doi:10.3390/ijms26188924)
Supplement: Supplementary file 1 [file ijms-26-08924-s001.zip › ijms-3798146-supplementary.pdf]

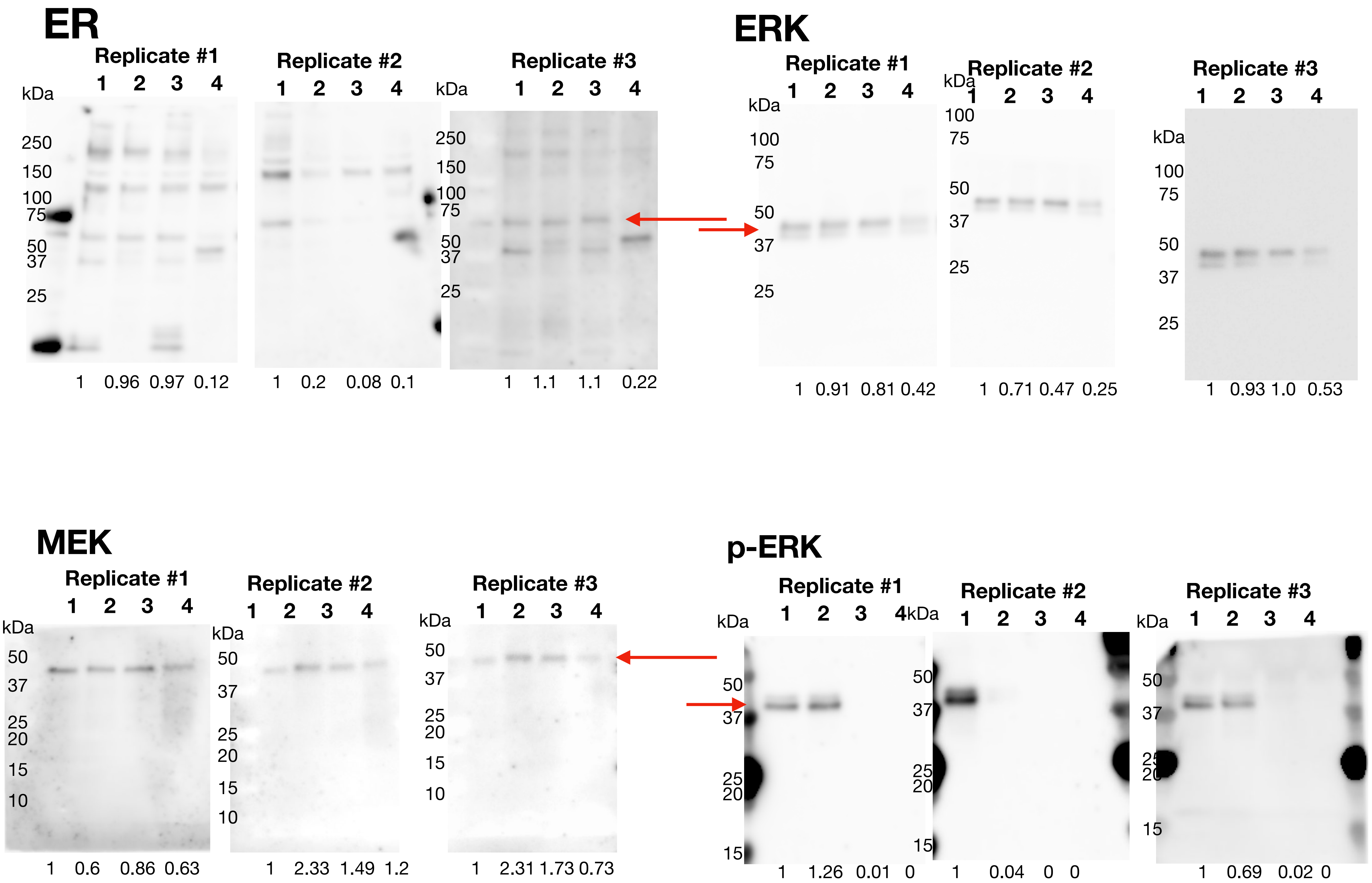

Legend
1: Control, 2: Fulvestrant, 3: MEK & FAK inhibitors, 4: Combination Fulvestrant with MEK and FAK inhibitors

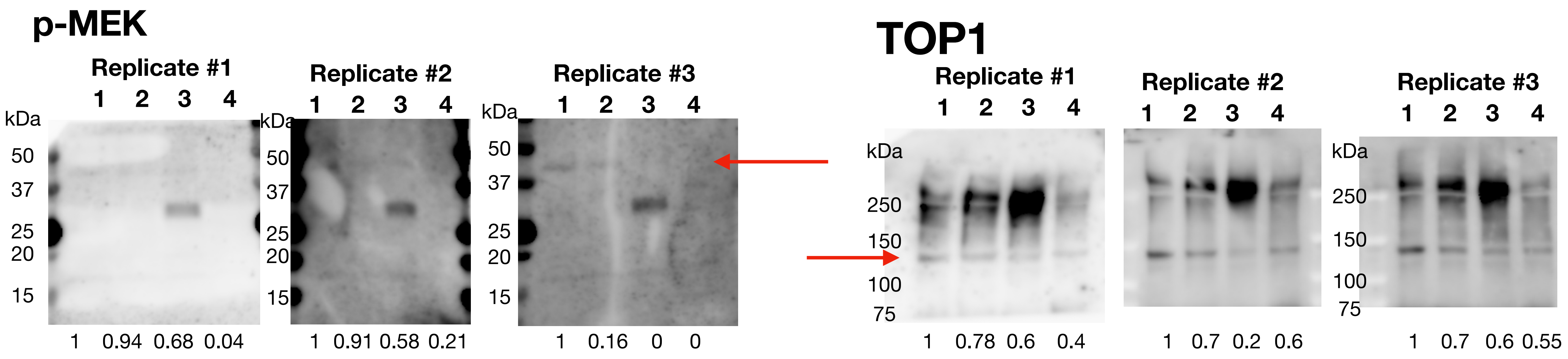

Legend
1: Control, 2: Fulvestrant, 3: MEK & FAK inhibitors, 4: Combination Fulvestrant with MEK and FAK inhibitors
